# Callose: a multifunctional (1, 3)-β–d-glucan involved in morphogenesis and function of angiosperm stomata

**DOI:** 10.1186/s40709-021-00150-9

**Published:** 2021-08-03

**Authors:** Panagiotis Apostolakos, Eleni Giannoutsou, Basil Galatis

**Affiliations:** Section of Botany, Department of Biology, National and Kapodistrian University of Athens, Athens, Greece

**Keywords:** Callose, guard cells, *Zea mays*, *Vigna sinensis*

## Abstract

**Background:**

Although the cellulose microfibril organization in guard cell (GC) walls play a crucial role in the mechanism of the stomatal function, recent work showed that matrix cell wall materials are also involved. Especially in the kidney-shaped stomata of the fern *Asplenium nidus*, callose actively participates in the mechanism of opening and closure of the stomatal pore.

**Scope:**

The present review briefly presents and discusses recent findings concerning the distribution and role of callose in the kidney-shaped stomata of the dicotyledon *Vigna sinensis* as well as in the dumbbell-shaped stomata of the monocotyledon *Zea mays*.

**Conclusion:**

The discussed data support that, in both categories of angiosperm stomata, callose is implicated in the mechanism of stomatal pore formation and stomata function by locally affecting the mechanical properties of the GC cell walls.

## Background

Guard cell (GC) morphogenesis and function, in both kidney and dumbbell-shaped stomata, is mainly based on the particular organization of cellulose microfibril arrays in cell walls, in particular in the periclinal ones, which is controlled by microtubule arrays radially aligned similarly to microfibrils [1–4]. Recently, data mostly derived from kidney-shaped stomata revealed that matrix cell wall materials, like different types of pectins and xyloglucans, are also involved in the mechanism of stomatal movement [2, 3, 5, 6]. The pectin network also seems to be important for the functional cell wall properties of the dumbbell-shaped stomata [7, 8].

The present article comments on the distribution and the possible functions of callose in the two types of angiosperm stomata. Callose is an interesting multifunctional (1, 3)-β-d-glucan, produced by callose synthases and degraded by β-1, 3-glucanases [9]. The data considered here have been mainly taken from a recent study on the kidney-shaped stomata of *Vigna sinensis* and the dumbbell-shaped ones of *Zea mays* [8]. Although, previous studies showed that callose plays an essential role in both morphogenesis and function of the kidney-shaped stomata of ferns [10], there is no information published on any other angiosperm species apart from *V. sinensis* and *Z. mays*.

### *Zea mays*

**Fig. 1:**
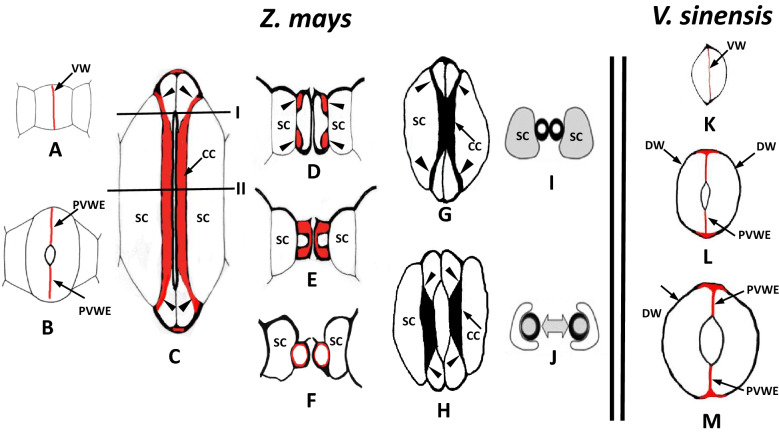
Diagrammatic representation of callose depositions in successive developmental stages of *Z. mays* (**A**–**J**) and *V. sinensis* (**K**–**M**) stomata. Newly formed (**A**), kidney-shaped (**B**) and dumbbell-shaped (**C**–**J**) stomata of *Z. mays* as well as newly formed (**K**), differentiating kidney-shaped (**L**) and mature kidney-shaped (**M**) stomata are shown. The cell wall regions rich in callose are marked in red. All the drawings represent stomata in paradermal view, except for (**D**–**F**) and (**I**), (**J**) that illustrate transverse stomatal sections. (**D**) shows a transverse view corresponding to the plane I of (**C**), while (**E**) a transverse view corresponding to the plane II of (**C**). The arrowheads mark the terminal thickenings of the central canal. (**G**) and (**H**) represent a closed and an open stoma respectively, in which callose is not depicted.* CC* central canal, * DW* dorsal wall,* PVWE* polar ventral cell wall end,* SC* subsidiary cell,* VW* ventral cell wall

In the newly formed stomata, callose has a prominent and relatively prolonged appearance in the young ventral wall (VW), which is the cell wall that separates GCs (Fig. 1A). Afterwards, it is deposited in the emerging GC cell wall thickenings. During stomatal pore formation, callose is removed from the cell walls delimiting the developing stomatal pore but persists at the polar VW ends, meaning the VW regions on both sides of the stomatal pore (Fig. 1B). As periclinal are defined the cell walls that are parallel to the epidermal surface, while as anticlinal, the cell walls that are vertical to it. In elongating and mature stomata, callose is localized at the polar VW ends, in the periclinal cell wall thickenings of the central canal and at the terminal thickenings of the central canal, meaning those that extend from the central canal towards the bulbous GC ends (Fig. 1C–E). The pattern of callose deposition in the central canal differs between open and closed stomata. In closed stomata, callose appears in the periclinal cell walls of the central canal. Notably, in the open ones, it is present not only in the periclinal cell walls, but also in the anticlinal ones that are shared between the GCs and the subsidiary cells (Fig. 1F; compare to Fig. 1E). Callose is also deposited at the transverse cell walls of the intervening cells of the stomatal row, in contact with the bulbous GC ends [8].

### *Vigna sinensis*

The VW of the newly formed stomata is rich in callose (Fig. 1K). In a similar way to *Z. mays*, in differentiating stomata, although callose is absent from the VW regions enclosing the developing stomatal pore, still persists at the polar VW ends (Fig. 1L). In addition, it impregnates the cell wall thickenings deposited at the junctions of the dorsal cell walls (Fig. 1L). The open mature stomata display the same pattern of callose distribution with that of the differentiating ones (Fig. 1M). In the functioning closed stomata, callose disappears from the major part of the polar VW ends but remains at their junctions with the dorsal cell walls [8].

## Callose and local GC cell wall thickenings

Callose deposition in GC cell wall thickenings seems to be a general characteristic of stomata. It has been found in the kidney-shaped stomata of dicotyledon plants and ferns [10], as well as in the monocotyledonous dumbbell-shaped stomata [8]. Callose enriches the cell wall thickenings in cotton fibers [11–13], tracheary cells [9, 14] and transfer cells [15]. It has been suggested that callose establishes a proper microenvironment for the local deposition of cellulose microfibrils and possibly of other cell wall materials, thus facilitating the formation of the local cell wall thickenings [8, 10, 11].

Shtein et al. [16, 17] showed that cellulose displays different degrees of crystallinity at different positions of the GC walls. According to the authors, the term “cellulose crystallinity” is used to define arrays of β-1,4 glycan chains, associated through numerous hydrogen bonds. Furthermore, these amorphous and crystalline domains of the cellulose microfibrils are further spatially organized into regions of differing crystallinity. In the kidney-shaped GCs, cellulose of high crystallinity has been detected at the polar stomatal ends. Ιn the dumbbell-like ones, crystalline cellulose is observed at the polar VW ends and in the central canal, including the terminal thickenings emerging from them. In both GC types, all the cell wall regions displaying high cellulose crystallinity were enriched by distinct local callose depositions (Fig. 1). The positional relationship between callose and high cellulose crystallinity is obvious in cotton fibers [11], tracheary cells [9] and characterizes the GCs of the fern *Asplenium nidus*. In the latter species, cellulose of high degree of crystallinity is deposited in the periclinal cell walls [16], where also prominent radial callose fibril systems are co-localized [18]. Ιt has been supported that callose creates a hydrated zone outside the plasmalemma, within which the cellulose microfibrils crystallize [9, 11, 13, 19]. However, the mechanism through which callose favors cellulose crystallinity still remains unknown. In *Asplenium nidus*, the radial callose fibrillar arrays in the periclinal cell walls are disassembled during stomatal opening and reappear during stomatal closure [10]. If this disappearance is followed by a change in the degree of cellulose crystallinity during stomatal movement, it will further support the existence of spatial and probably functional relationship between callose and cellulose crystallinity.

## Callose and stomatal pore formation

The schizogenous stomatal pore formation in angiosperms is a phenomenon concomitant to GC morphogenesis, involving two processes: the weakening of the middle lamella of the VW and the application of mechanical forces generated during assumption of the permanent kidney shape in *V. sinensis* and the temporary one in *Z. mays* GCs [1]. These forces disrupt the periclinal cell walls and separate the VW partners of the GC pair at the stomatal pore site. Notably, the middle lamella of the young VW of *Ζ. mays* and *V. sinensis*, in contrast to that of the lateral cell walls of the former and to that of the dorsal cell walls of the latter, appears electron transparent in TEM micrographs. In addition, it gives a negative reaction to Thiery’s test that is specific for insoluble polysaccharides, apart from cellulose and callose [20, 21]. Recently, Rui et al. [22] concluded that in the dicotyledonous *Arabidopsis thaliana* ‘‘homogalacturonans delivery and modification, and guard cell pressurization, make functional contributions to stomatal pore initiation and enlargement’’ and that homogalacturonan degrading enzymes are locally activated in the region of the stomatal pore.

In Z. *mays* and *V. sinensis* stomata callose has a prolonged appearance in young VW that is followed by the progressive local callose removal from the cell walls of the developing stomatal pore (Fig. 1B; compare to Fig. 1A and L; compare to Fig. 1K). Obviously, the callose degrading enzyme is locally activated. Callose degradation from the cell walls delimiting the forming stomatal pore, possibly facilitates the detachment of the adjoined VW partners at this specific region. This can be understood considering the stiffening property of callose on the cell wall, since its presence allows the cell wall to resist in tension and compression stresses [23]. In contrast, callose persists at the polar VW ends (Fig. 1B, L). Its maintenance at these sites may also constrain the detachment of the cell wall strictly at the median region of the VW, preventing the expansion of the stomatal pore towards the stomatal ends. A similar activity has been attributed to callose during development of the intercellular spaces at the mesophyll [24, 25]. This callose behaviour favours the view that callose participates in the mechanism of stomatal pore formation in angiosperms, a phenomenon well documented in the fern *Asplenium nidus* [10, 26].

## Possible callose involvement in stomatal movement

The radial callose fibrillar arrays deposited in the periclinal cell walls of the kidney- shaped GCs of the fern *Asplenium nidus*, which are co-aligned with the radial cellulose microfibrils, possibly participate in stomatal opening, reinforcing the role of the latter in the tangential periclinal GC wall expansion that is critical for stomatal opening [10, 27]. This suggestion has been experimentally supported [27].

Callose is absent from the periclinal cell walls of the functional kidney–shaped GCs of the dicotyledonous *V. sinensis*. It is localized at the polar VW ends as well as in the thickenings deposited at the junctions of the dorsal cell walls (Fig. 1 L, M). It is well known that during stomatal opening, the polar VW ends of the kidney-shaped stomata are under intense mechanical stress [3, 17, 28, 29]. Callose, functioning as a stiffening material, probably strengthens the polar VW ends to withstand the mechanical forces generated during stomatal opening [8]. If the VW ends are not stiff enough, the stomatal pore will not open successfully.

Gensler [19] considering the presence of callose in the periclinal cell walls of the kidney-shaped GCs of the fern *Asplenium nidus* [18, 27] has assumed that callose creates a protonic electrical circuit at the periclinal cell walls of the GCs. Such a circuit would facilitate proton transfer between different parts of the VW and further provide a driving force for concomitant potassium ion entry and exit between GCs and subsidiary cells. In this way, callose could participate in the stomata function mechanism. However, this view cannot be applied in kidney-shaped GCs of *V. sinensis*, because callose is absent from their periclinal cell walls.

Although both the dicotyledonous *V. sinensis* and the fern *A. nidus* have kidney-shaped stomata, callose displays a different distribution pattern between the two. It implies that it plays a different role during stomatal movement in them. In *V. sinensis*, callose is absent from the periclinal cell walls of the GCs [8], while it is present at these cell walls of *A. nidus* GCs [18, 27]. These differences may be related not only to the particular morphology of the GCs but also to the chemical composition of the cell walls of the GCs of these two plants. In *A. nidus*, the GCs display swollen polar ends facing the substomatal cavity and intense cell wall thickenings at the junction sites of the polar VW ends with the external periclinal cell wall [30]. These cell wall regions are traversed by many cellulose microfibrils arranged parallel to the epidermal surface [30]. These structural features are absent from the GCs of *V. sinensis*. Furthermore, while the GC walls of the dicotyledonous, as *V. sinensis*, are rich in pectins [8, 17], they seem to participate in a lower degree in the cell wall composition of *A. nidus* GCs [17]. According to Shtein et al. [16, 17], the ferns, including *A. nidus*, use crystalline cellulose as a localized strengthening material in the central region of the GCs that participates in stomatal movement. This notion is further supported by the presence of callose at the exact same sites. On the contrary, in dicotyledonous stomata, the role of crystalline cellulose and callose is probably served by pectins located at the respective regions [17]. Nevertheless, in *A. nidus* stomata too, the callose deposited at the cell wall thickenings of the polar VW ends [18, 27] probably reinforces the specific regions in order to withstand the mechanical forces exerted during stomatal opening, as it has already been suggested in *V. sinensis* stomata [8].

During opening of the dumbbell-shaped grass stomata, the bulbous GC ends swell and become deformed. The radial cellulose microfibrils in both the periclinal cell walls of the bulbous GC ends, which diverge from the edge of the central canal towards their ends [20] and the pairs of terminal central canal thickenings, seem to control the pattern of expansion and deformation of the bulbous GC ends. The swelling of the bulbous GC ends appears asymmetrical, being more intense towards the VW than towards the dorsal cell wall (Fig. 1H; compare to Fig. 1G). The mechanical forces generated by the elevated GC turgor are finally exerted on both the polar VW ends and on the terminal canal thickenings, to induce stomatal opening. It is achieved when the central canals are displaced to some extent ‘‘into the subsidiary cells’’ [31, 32] (Fig. 1G; compare to Fig. 1H). When the bulbous ends of the GCs swell, the terminal cell wall thickenings that emerge from the central canal and enter the junctions of the periclinal cell wall with the lateral ones in the bulbous GC ends (arrowheads in Fig. 1) probably enforce the central canals to move towards the subsidiary cells, acting like “levers”. This becomes possible because the central canal and the terminal cell wall thickenings in each GC constitute a united system [20]. At the same time mechanical forces applied on the VW ends also contribute to the lateral displacement of the central canals.

The development of this particular mechanism of stomatal movement became probably necessary because of the unique morphology of the dumbbell-shaped stomata of the Poaceae. Usually, in *Z. mays*, the junctions of the VW with the transverse cell walls display large gaps, through which cytoplasm, plastids and mitochondria can move from one GC to the other [20]. However, this VW discontinuity enables GCs to be synchronized and to function as one cell during stomatal movement. In addition, the osmotic and turgor pressure ‘‘synchronization’’ between the GCs and the subsidiary cells, ‘‘see-sawing’’ according to Franks and Farquhar [31], is also functionally important. The increase of the GC turgor, keeping pace with the decrease of that of the subsidiary cells makes feasible the change of the shape of the latter cells to ‘‘accept the lateral central canal displacement’’ [31, 32] (Fig. 1F; compare to Fig. 1E and  J; compare to Fig. 1I). As Franks and Farquhar [31] and Nunes et al. [33] pointed out, the four-celled dumbbell-shaped stomatal complexes of Poaceae, due to their unique structure, became able to attain wider pore apertures and faster response to environmental changes than any other stomatal type.

Callose enrichment of the central canal cell walls as well as those of bulbous GC ends (Fig. 1C–E), increases their stiffening making them rigid enough to secure the lateral displacement of the central canal ‘‘into the subsidiary cells’’ [8]. Especially, callose deposition in the terminal thickenings of the central canal increases their stiffness to fulfil the critical role in stomatal opening suggested above. The endings of the cell wall thickenings of the central canal display high degree of cellulose crystallinity [28], so Rui et al. [6] concluded that the cell wall in these regions display intense stiffness. Obviously, this stiffness is further increased by the presence of callose. Callose detection at the cell wall of the intervening cell adjacent to the polar end of the open stomata [8] is probably a response to mechanical forces exerted on it during increase in volume/deformation of the bulbous GC ends. In addition, the presence of callose in the lateral GC cell wall of the central canal, (Fig. 1F), may have either a sealing function for the preservation of GC and/or the subsidiary cell turgor or more possibly it is formed as the result of mechanical stresses imposed on this cell wall during the lateral movement of the GC central canal. The above consideration allows the suggestion that the extensive GC callose depositions significantly reinforce the rigidity of the dumbbell-shaped stomata during stomatal opening and closure.

## Conclusions

The above overview allows the hypothesis that callose is implicated not only in morphogenesis of the angiosperm stomata but also in their function. It appears in GC cell walls in an accurately spatially and temporarily controlled manner, in order to perform specific functions. In the functioning angiosperm stomata, callose constitutes a dominant matrix of the polar VW ends and/or the local GC cell wall thickenings. Although it seems likely that the described pattern of distribution as well as the suggested role(s) of callose should represent general characteristics of the angiosperm stomata, further studies are needed to verify them. Moreover, since the mechanics of the dumbbell-shaped stomata has not been adequately studied, further work should be carried out to understand the mechanical aspect of their movement. Evidently, the unique structure of the dumbbell-shaped stomata has led to the development of a specific mechanism of stomatal movement, deviating from that of the kidney-shaped ones.

## Data Availability

The data sets analyzed during the current study are available from the corresponding author on reasonable request.
